# Genome diversification of symbiotic fungi in beetle-fungus mutualistic symbioses

**DOI:** 10.1093/ismejo/wraf039

**Published:** 2025-02-27

**Authors:** Yin-Tse Huang, Khaled Abdrabo El-Sayid Abdrabo, Guan Jie Phang, Yu-Hsuan Fan, Yu-Ting Wu, Jie-Hao Ou, Jiri Hulcr

**Affiliations:** Department of Biomedical Science and Environmental Biology, Kaohsiung Medical University, Kaohsiung 80708, Taiwan; Department of Medical Research, Kaohsiung Medical University Hospital, Kaohsiung 80708, Taiwan; Department of Biomedical Science and Environmental Biology, Kaohsiung Medical University, Kaohsiung 80708, Taiwan; Botany and Microbiology Department, Faculty of Science, Assiut University, Assiut 2074020, Egypt; Department of Biomedical Science and Environmental Biology, Kaohsiung Medical University, Kaohsiung 80708, Taiwan; Department of Biomedical Science and Environmental Biology, Kaohsiung Medical University, Kaohsiung 80708, Taiwan; Department of Biomedical Science and Environmental Biology, Kaohsiung Medical University, Kaohsiung 80708, Taiwan; Department of Forestry, National Pingtung University of Science and Technology, Pingtung 91201, Taiwan; Tohoku Agricultural Research Center, National Agriculture and Food Research Organization, Morioka, Iwate 020-0198, Japan; School of Forest, Fisheries, and Geomatics Sciences, University of Florida, Gainesville, FL 32603, United States; Department of Entomology and Nematology, University of Florida, Gainesville, FL 32603, United States

**Keywords:** ambrosia fungi, symbiosis, comparative genomics, CAZymes, phylogenomic analysis

## Abstract

Ambrosia beetles and their fungal symbionts represent a widespread and diverse insect-fungus mutualism. This study investigates the genomic adaptations associated with the evolution of the ambrosia lifestyle across multiple fungal lineages. We performed comparative genomic analyses on 70 fungal genomes from four families (*Irpicaceae, Ceratocystidaceae, Nectriaceae*, and *Ophiostomataceae*), including 24 ambrosia and 34 nonambrosia lineages. Our phylogenomic analyses reveal multiple independent colonization of insect vectors by the fungi, spanning from the mid-Cretaceous (114.6 Ma) to the early Quaternary (1.9 Ma). Contrary to expectations for obligate symbionts, ambrosia fungi showed no significant genome-wide modification in size, gene count, or secreted protein repertoire compared to their nonsymbiotic relatives. Instead, we observed conservation of most assessed genomic features; where genome traits differ between free-living relatives and ambrosia fungi, the changes are lineage-specific, not convergent. Key findings include lineage-specific expansions in carbohydrate-active enzyme families (AA4 in *Nectriaceae*, CE4 in *Ophiostomataceae*, and GH3 in *Ophiostomataceae* and *Ceratocystidaceae*), suggesting potential enhancement or loss of lignin modification, hemicellulose deacetylation, and cellulose degradation in different ambrosia lineages. Repeat-induced point mutation analysis revealed family-specific patterns rather than lifestyle-associated differences. These results highlight the diverse genomic strategies employed by ambrosia fungi, demonstrating that symbiont evolution can proceed through refined, lineage-specific changes rather than genome-wide or convergent alterations. Our genomic analyses do not reveal patterns typically associated with domestication in these ambrosia fungi, suggesting they may represent free-living fungi that co-opted wood-boring beetles as vectors through subtle, lineage-specific adaptations.

## Introduction

Insect-fungal symbioses represent stable evolutionary strategies with ecological significance in nutrient cycling and wood decomposition. The ambrosia symbiosis—most widespread, diverse, and repeatedly evolved—involves wood-boring beetles (Scolytinae and Platypodinae) and fungal partners from various orders. Beetles transport fungi between dead trees and feed on fungal conidia, while fungi benefit from access to new hosts. This mutualism demonstrates co-evolution: beetles developed mycangia to carry fungi [1], while fungi adapted ambrosial cells for beetle nutrition [2, 3], with both partners evolving specialized interdependent adaptations.

Both ambrosia beetles and fungi exemplify convergent evolution [1, 4], with the mutualism independently evolving 12-16 times in beetles [4, 5] and over 10 times across both Basidiomycota and Ascomycota fungi [6, 7]. These multiple instances of parallel evolution offer unique opportunities to study genomic adaptations supporting mutualism across diverse evolutionary backgrounds.

Recent genomic studies on various fungal symbionts outside of the beetle system, including arbuscular mycorrhizal fungi and fungal crops of insects, have revealed insights into genome evolution and functional gene diversification [8–12]. However, most such symbioses evolved only once or a limited number of times within their respective phylogenies. Ambrosia fungi and their multiple evolutionary origins offer unparalleled designs for comparative phylogenetic analyses.

Despite apparent lifestyle uniformity, metabolomic profiles of ambrosia fungi do not converge across lineages, suggesting the uniqueness of each ambrosial system [2, 13]. Unlike internal symbionts, ambrosia fungi spend most of their lifecycle outside the animal host in their ancestral niche (freshly dead trees). Consequently, their genomes likely won't show major metabolic differences compared to free-living relatives. They can use wood polymers as carbon sources but generally remain non-competitive lignocellulose degraders compared to true wood decay fungi [14, 15].

Key fungal adaptations include mycangium colonization and nutrient sequestration for beetle development, which is often associated with enlarged ambrosial cells suitable for beetle nutrition. No robust theory predicts which metabolic pathways would be regulated for these adaptations, which might be subtle and varied across fungal lineages due to multiple independent origins and subsequent beetle-specific specialization [1, 16].

Fungal genomes could reveal multiple adaptations to the symbiotic lifestyle through various specialized elements: functional genes encoding carbohydrate-active enzymes (CAZymes), lipases, peptidases, and cell wall-degrading enzymes serve as crucial indicators of metabolic capabilities [17], with recent research suggesting enzymatic profiles differ from previous assumptions—some groups (*Phialophoropsis, Irpex*) significantly degrade wood [18] while others specialize in rapid utilization of simple gallery nutrients [15, 16]; G-protein coupled receptors (GPCRs) enable environmental sensing of nutrients, pheromones, and stress factors [19], with certain families like Pth11-like receptors enabling host recognition in pathogens [20] but having unexplored roles in symbiotic fungi; candidate secreted effector proteins likely mediate host interactions [21, 22] by facilitating nutrient exchange and modulating immune responses—particularly important for mycangium colonization; and Repeat-Induced Point mutation (RIP), typically associated with sexual reproduction, potentially influences genome architecture through cryptic sex despite ambrosia fungi being primarily asexual [23, 24], as suggested by sporadic sexual state discoveries [25, 26].

Our study explores the genomic foundations underlying ambrosia fungi’s adaptation to their specialized ecological niche. We compared 70 fungal genomes across four families (*Irpicaceae, Ceratocystidaceae, Nectriaceae*, and *Ophiostomataceae*), including 24 ambrosia and 34 nonambrosia lineages, plus 12 outgroups. This dataset, comprising 8 newly sequenced and 62 retrieved genomes, represents independent origins of ambrosia fungi. Our research aims to uncover distinctive genomic characteristics associated with the ambrosia lifestyle by addressing four key questions: (i) How do the genomic features of ambrosia fungi compare to those of other fungal symbionts and their nonsymbiotic relatives? (ii) What specific enzymatic adaptations correlate with the origins of the symbiotic relationship with ambrosia beetles? (iii) What is the correlation between secreted proteins and GPCRs with mutualistic recognition in ambrosia symbiosis? (iv) What role does RIP mutation play in shaping the genome architecture of ambrosia fungi? This study explores the evolutionary patterns, genomic foundations, and ecological adaptations of ambrosia fungi, advancing our understanding of insect-fungal symbiosis from mere observations to genome-level understanding.

## Material and methods

### Fungal strain selection and preparation

The fungal strains used in this study (Supplementary Table S1) represent a spectrum of ecological associations within the ambrosia beetle system. Based on comprehensive literature reviews [1, 27, 28] and exhaustive searches of public genome repositories (NCBI Genome database and JGI MycoCosm), our dataset represents, to the best of our knowledge, all currently available genome sequences of ambrosia fungi. We defined ambrosia fungi as dominant and consistent mycangial isolates, as mycangial presence indicates successful adaptation to both transport within and colonization of these specialized beetle structures [29]. In the analyses, we paired these with nonambrosia counterparts selected based on phylogenetic relatedness and ecological relevance. The nonambrosia fungi were isolated either from ambrosia beetle body surfaces or from associated wood substrates. This approach allowed for comparative genomic analysis across different degrees of symbiotic association within similar ecological niches.

### DNA extraction and quality assessment

DNA was extracted from 10-day-old fungal cultures. Fungal tissues were scraped from potato dextrose agar (PDA) medium, placing in microcentrifuge tubes with zirconia beads, and disrupting mycelium via Vortex-Genie 2 at 3200 rpm for 4 minutes. DNA purification used modified bio-on-magnetic beads (BOMB) protocol [30] with the ZiXpress 32 automated system, and quantification was performed using Invitrogen Qubit Fluorometer.

### Genome sequencing, assembly, and quality assessment

Genomic DNA was sequenced using Oxford Nanopore Technology with Native Barcoding Kit 24 V14 per manufacturer's protocol. We loaded 0.4 μg DNA library onto an R10.4.1 flow cell and sequenced on MinION using MinKNOW v23.07.5. Raw outputs were basecalled with dorado v0.5.1 (SUP model), then processed through nanoACT pipeline: quality-filtering (q-score=9, length 500-1,000,000), demultiplexing with singlebar(), and trimming with trim_reads(). Trimmed reads were assembled using Flye v2.9.3 with default settings and “--nano-hq” option. Consensus sequences of the assemblies were polished using Medaka v1.8.1 https://github.com/nanoporetech/medaka). Transposable element (TE) coverage in the genomes was identified using RepeatModeler v2.0.5 and RepeatMasker v4.1.6. Genome completeness was assessed using BUSCO v5.6.1 with fungi-odb10 reference and default parameters. The resulting genome assemblies were deposited in NCBI under Bioproject accession PRJNA1084195 (Supplementary Table S1).

### Comparative genomic feature analyses

We performed genome annotation using BRAKER3 v3.0.5, which integrates RNA-Seq data from the Sequence Read Archive with gene predictions from AUGUSTUS and GeneMark-ET. Gene functions were subsequently assigned using eggNOG-mapper v2.1.12 with the eggNOG orthology v5.0.2 database.

To predict the fungal secretome, we annotated our genomes using multiple databases. We identified signal peptides using SignalP 6.0 [31], transmembrane domains using TMHMM via Galaxy [32], and subcellular localization using TargetP [33]. Proteins meeting any of these criteria were included in the predicted secretome. We further characterized the secretome for lipases and peptidases by aligning protein sequences to the Lipase Engineering Database [34] and MEROPS peptidase database [35] using DIAMOND v2.1.8 [36]. The top hit for each protein was selected based on bitscore. CAZymes were annotated using dbCAN3 [37] with HMMER, Diamond, and dbCAN_sub for CAZyme family annotation. Specifically, we only included annotated CAZymes genes that were concurrently annotated by both HMMER and dbCAN_sub. We categorized CAZymes into Plant Cell Wall-Degrading Enzymes (PCWDEs) following the classification scheme of Miyauchi *et al.* [8]. GPCRs were identified through a BLASTP search against the best hits in the GPCRDB database [38], and their functional roles were cross-referenced with UniProt [39]. To identify candidates for secreted effector proteins (CSEPs), we used Effectorp 3.0 [40]. We retained both apoplastic and cytoplasmic effectors in our analysis, excluding only those identified as “non-effector” by the tool. All analyses were performed using default parameters unless otherwise specified.

We compared gene copy numbers between ambrosia and non-ambrosia fungi using Wilcoxon rank-sum tests via R's wilcox.test(), calculating mean gene numbers for each group and evaluating statistical significance of differences. MANOVA (manova() in R) was used to examine how ambrosia lifestyle and fungal family affected diversity indices (gene richness and Shannon entropy) across functional gene categories, using Pillai's trace statistic for significance testing. Key genomic features (genome size, TE coverage, gene count, scaffold N50, secreted protein count, and GC content) were compared using Phylogenetically Independent Contrasts (PIC) with ape and phangorn R packages to control for phylogenetic non-independence.

### Repeat-induced point mutation estimation

We examined Repeat-Induced Point (RIP) mutations in repeat-masked genomes of ambrosia and non-ambrosia fungi using the RIPper software [41] with stringent parameters (RIP product > 1.1, RIP substrate ≤ 0.75, composite index > 7), employing 1,000 bp sliding windows with 500 bp steps. RIP-associated genes (DIM-2, RID, HP-1, DIM-5, DIM-7, CUL4, DDB-1) were identified via BLASTp against Neurospora crassa homologs (E-value cutoff 1e-5). Mann-Whitney U tests compared RIP metrics between fungal groups, analyzing: number of RIP-affected windows, genomic proportion affected by RIP, and count of Large RIP Affected Regions (LRARs), both across all fungi and within individual families. Heatmaps created with seaborn and matplotlib visualized RIP class (ranging from Class 1 [0-0.2%] to Class 6 [≥20%] according to [42]), standardized RIP features, and gene presence/absence across species, with RIP features standardized using sklearn's StandardScaler.

### Phylogenetic reconstruction

We reconstructed fungal phylogeny using representative genomes across Basidiomycota and Ascomycota (including species closest to ambrosia fungi) with two Mucoromycota outgroups. BUSCO v5.5.0_cv1 with fungi_odb10 database generated single-copy orthologs from each genome. Sequences were aligned with MAFFT v7.490 using default parameters, then trimmed with ClipKIT v2.3.0 using “smartgap” option. We retained BUSCO gene alignments with ≥90% taxon occupancy. ModelTest-NG v0.1.7 selected optimal evolutionary models for each gene. For species tree reconstruction, 742 retained BUSCO gene alignments were concatenated and partitioned by gene using the best models from ModelTest-NG. Trees were built using IQ-TREE2 v2.0.7 with parameters “-bb 1000 -alrt 1000” and rooted to Mucoromycota outgroups in FigTree. For gene trees, each single-copy ortholog alignment was analyzed separately using IQ-TREE2 with independently selected evolutionary models, then all resulting trees were concatenated. ASTRAL v5.7.1 [43] was then employed to infer individual phylogenies from these *BUSCO* genes.

To assess gene tree-species tree discordance beyond incomplete lineage sorting (ILS), we performed chi-square tests comparing observed vs. expected distributions of concordant/discordant topologies. Using IQ-TREE, we calculated gene concordance factor (gCF) and site concordance factor (sCF) for each branch in the species tree. We then compared observed concordant vs. discordant gene and site numbers to the expected 1/3 ratio under ILS using a custom R function (analysisR_updated.ipynb). Branches with *P* values < 0.05 for either gCF or sCF were identified as significantly discordant, potentially resulting from processes beyond ILS, such as introgression or incomplete taxon sampling.

### Divergence dating

To estimate evolutionary transitions to ambrosial lifestyle timing, we used RelTime in MEGA X [44], which calculates relative divergence times by leveraging molecular rates across phylogenetic branches [45, 46]. We analyzed concatenated alignments of single-copy BUSCO orthologs and ML topologies from phylogenetic reconstruction. Three calibration nodes were incorporated: Ascomycota/Basidiomycota divergence (582-749 Ma) [47], Sclerotiniaceae/Sordariomycetes split (239.7-320 Ma), and fossil record constraining Diaporthales minimum age (>136 Ma) [48], with the latter aligning with Vanderpool et al. (2018) approach [49]. Molecular data was modeled using Tamura-Nei model with gamma-distributed rates (TN93+G), selected as best fit based on Bayesian Information Criterion scores.

### Ancestral state reconstruction

We performed ancestral state reconstruction (ASR) to trace ambrosia status evolution across our 61-taxa phylogeny using maximum likelihood and stochastic character mapping approaches in R with ape and phytools packages [50]. For maximum likelihood reconstruction, we utilized ace() from ape with the equal rates (ER) model, estimating marginal ancestral state likelihoods at all nodes based on current states only. To address uncertainty, we implemented stochastic character mapping using make.simmap from phytools, which employs MCMC to sample 1000 character histories from their posterior probability distribution using the ER model. Results were visualized on the phylogenetic tree with pie charts at internal nodes showing either maximum likelihood probabilities of each ancestral state or the proportion of stochastic simulations supporting each state, summarizing the 1000 character histories.

## Results and discussion

### Genome assembly and quality assessment

**Figure 1:**
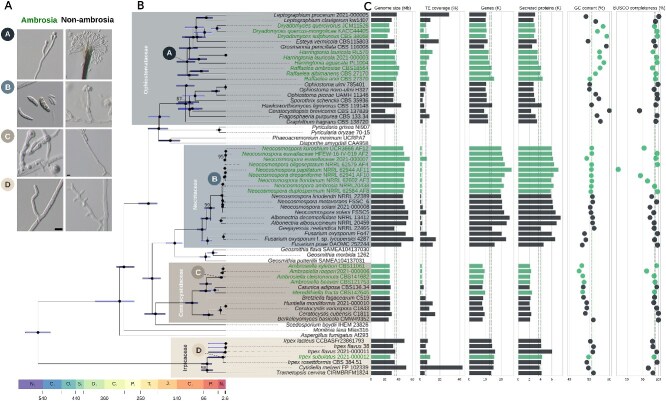
Evolution and genomic features of ambrosia fungi. (A) Representative fungal species illustrate morphological differences (enlarged cells) in ambrosia vs. nonambrosia fungi; colored circles denote representative species in four fungal families. (B) Dated consensus phylogenomic tree showing multiple origins of ambrosia fungi across Ascomycota and Basidiomycota. Green taxa represent ambrosia fungi. Nodes with support values less than 100 are noted for reference; all other nodes have a support value of 100. Time scale in millions of years ago (Ma) is shown at the bottom. (C) Comparison of key genomic features (genome size, TE coverage, gene count, and secreted protein count) between ambrosia (green) and nonambrosia (black) fungi; dotted lines present median value of GC content (%) and BUSCO completeness (%) of ambrosia (green) and nonambrosia (black). Outgroup species not shown.

We sequenced genomes of eight species in the *Ophiostomataceae, Ceratocystidaceae, Nectriaceae*, and *Irpicaceae* families, including both ambrosia and nonambrosia fungi, and obtained genomes of 62 additional fungal species from the NCBI Genome database. Genomic features are summarized in Supplementary Table S2. All genomes showed BUSCO values above 96%, indicating high-quality assemblies (Fig. 1). Genome sizes varied across families, ranging from 28 Mb (*Ambrosiella roeperi* T.C. Harr. & McNew) to 54.4 Mb [*Neocosmospora euwallaceae* (S. Freeman, Z. Mendel, T. Aoki & O’Donnell) Sand.-Den., L. Lombard & Crous)]. GC content was relatively consistent [45% in *A. roeperi* to 55.3% in *Harringtonia lauricola* (T.C. Harr., Fraedrich & Aghayeva) Z.W. de Beer & M. Procter]. Assembly contiguity varied considerably, with N50 values ranging from 0.38 Mb [(*Irpex subulatus* (Ryvarden) Z.B. Liu & Y.C. Dai)] to 4.45 Mb (*N. euwallaceae*). Scaffold counts ranged from 39 [(*Leptographium procerum* (W.B. Kendr.) M.J. Wingf., Trans)] to 476 (*I. subulatus*). Protein-coding gene counts showed substantial variation, from 8158 [(*Huntiella moniliformis* (Hedgc.) Z.W. de Beer, T.A. Duong & M.J. Wingf.)] to 21 224 (*N. euwallaceae*). *Nectriaceae* family members [(*N. euwallaceae* and *Neofusicoccum solani* (Mart.) L. Lombard & Crous)] exhibited higher gene counts compared to other families.

**Figure 2:**
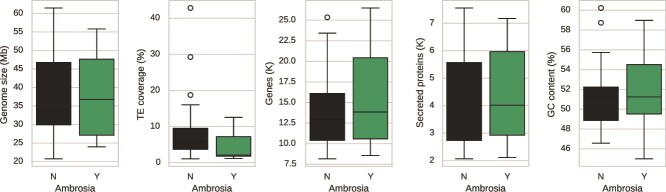
Statistical analysis of genomic features shown in Fig. 1C between ambrosia (green) and nonambrosia (black) fungi. No statistically significant differences were found for genome size, TE coverage, gene count, secreted protein count, or GC content across all species tested.

No consistent genomic patterns (Fig. 2) were observed between ambrosia and nonambrosia fungi across families, implying that the ambrosia lifestyle may not be associated with convergent genome-wide characteristics.

### Multiple symbiosis events of ambrosia fungi across Dikarya

Our phylogenomic analyses, based on genome-wide sequence data from 70 taxa—including 24 ambrosia fungi, 34 nonambrosia relatives, two *Mucoromycota* outgroups (not shown in the time-calibrated phylogeny), and ten taxa from the *Eurotiomycetes, Leotiomycetes*, and *Sordariomycetes* groups—enhanced the resolution of the phylogenetic topology. The resulting phylogenomic tree indicates that ambrosia fungi colonized the insect vectors repeatedly over a wide evolutionary period, spanning from the mid-Cretaceous (114.6 Ma) to the early Quaternary (1.9 Ma) (Table 1). The tree highlights multiple independent beetle colonization events and subsequent co-diversification, aligning with patterns reported in prior studies (Fig. 1 and Supplementary Fig. S1).

**Table 1:** Divergence dates of ambrosia lineages in the present study.

Ambrosia species	Family	Crown age	CI lower	CI upper	Stem age
*Dryadomyces* spp.	*Ophiostomataceae*	66.4	44.1	100.1	102.0
*Harringtonia* spp.	*Ophiostomataceae*	84.7	60.2	119.1	113.5
*Raffaelea* spp.	*Ophiostomataceae*	114.6	86.4	152.0	121.3
*Neocosmospora* spp.	*Nectriaceae*	9.4	5.8	15.1	11.8
*Ambrosiella* spp.	*Ceratocystidaceae*	14.3	9.9	20.7	60.1
*Meredithiella* spp.	*Ceratocystidaceae*	34.6	23.0	52.1	60.1
*Irpex subulatus*	*Irpicaceae*	1.9	1.6	54.2	9.2

### Ancient origins and asynchronous evolution in *Ophiostomataceae*

The phylogenetic topology of *Ophiostomataceae* in our study largely aligns with previous findings, albeit with some discrepancies. Our placement of *Graphilbum fragrans* (Math.-Käärik) Z.W. de Beer, Seifert & M.J. Wingf. as the sister lineage within the *Ophiostomataceae* supports the findings of Nel *et al.* [51] and Vanderpool *et al.* [49]. This differs from the topology presented in de Beer *et al.* [52], where *G. fragrans* is nested with *Leptographium, Dryadomyces*, and *Esteya* species. Consistent with earlier studies, our phylogeny indicates *Raffaelea* sensu stricto species clustered with a clade comprising *Leptographium, Dryadomyces, Harringtonia, Esteya*, and *Grosmannia* species, albeit with moderate support. Although our analysis yielded 100% bootstrap support for this grouping, it is important to note that concatenated supermatrix datasets can sometimes artificially inflate support values, potentially masking underlying uncertainties [53]. gCF/sCF for this node (6.68/34 respectively) closely mirror those reported by de Beer *et al.* [52] (10.1/34.6). Our ILS tests (Supplementary Table S3) revealed significant values for both gCF (*P* = 3.85E-04) and sCF (*P* = 4.27E-07), suggesting that introgression or incomplete taxon sampling may have influenced this branching pattern. Future studies incorporating more closely related species are needed to provide a better resolution of their evolutionary relationships. Consistent with previous research, our phylogeny also shows a well-supported clade comprising *Ophiostoma, Sporothrix, Fragosphaeria*, and *Ceratocystiopsis*.

The *Ophiostomataceae* exhibit variable degrees of adaptation to beetles, characterized by high levels of promiscuity in beetle host associations [54, 55]. Our latest estimates suggest earlier divergence times compared to previous studies, pushing the origin of the first ambrosia symbioses deeper into the past.

The *Dryadomyces* clade (corresponding to the *Raffaelea sulphurea* complex or RS clade in Vanderpool et al., 2018) diverged approximately 66.4 million years ago (Ma); (95% CI: 44.1–100.1 Ma), with a stem age of 102 Ma. This is considerably earlier than the 33 Ma crown age and 58 Ma stem age previously reported. The *Harringtonia* clade (equivalent to the complex previously named after *Raffaelea lauricola* or RL clade) shows an even older divergence at 84.7 Ma (95% CI: 60.2–119.1 Ma), surpassing the previous estimate of 67 Ma for its crown age. The *Raffaelea* sensu stricto clade (corresponding to the *Raffaelea ambrosiae* complex or RA clade) exhibits the earliest divergence at 114.6 Ma (95% CI: 86.4–152 Ma), with a stem age of 121.3 Ma. This substantially predates the 86 Ma crown age previously reported. It also aligns closely with estimates of the origin of *Platypodinae* (*Coleoptera, Curculionidae*), the oldest clade of ambrosia beetles, at ~96 Ma by Jordal [56], but it predates the estimate by Shin *et al.* [57].

These deep divergence times, particularly in the *Raffaelea* clade, suggest that the lineages leading to these ambrosia fungi were already distinct around or before the estimated origin of fungus-feeding in some beetle groups. Although beetle mycangia morphology has been suggested to influence symbiont fidelity [27, 54, 58], the frequent colonization of different ambrosia beetle lineages by *Ophistomataceae* may reflect aspects of fungal ecology rather than beetle morphology [59]. Some fungal traits that are beneficial or conducive to transmission within the ambrosia symbiosis may have evolved prior to their association with beetles, predisposing these fungi to readily establish symbiotic relationships [60]. This is consistent with the observation that different beetle lineages may have been independently colonized by various *Ophiostomataceae* fungi at different times, leading to the observed pattern of asynchrony. This revised evolutionary scenario provides a more nuanced understanding of the complex and ancient nature of these symbiotic relationships, emphasizing the importance of fungal preadaptations in directing the ambrosia symbiosis.

### Specialized symbioses and evolutionary synchronization in *Ceratocystidaceae*

In contrast to the promiscuous nature of *Ophiostomataceae, Ceratocystidaceae* fungi exhibit higher fidelity to their beetle vectors. The *Ambrosiella*-*Meredithiella* clade diverged 34.6 Ma (95% HPD: 22.9–52.1 Ma), with crown ages closely approximating the origins of their respective hosts (Xyleborini and *Corthylus*) [61].

The *Ceratocystidaceae* is a promising model for comparative evolutionary analysis of the symbiosis evolution. Several genera of ambrosia fungi evolved independently in this clade, and all are highly specific to their beetle vectors (unlike many of the *Ophistomataceae*) [26, 27, 29]. The fungus-beetle evolutionary units differ slightly in their relationships to host trees. Species within the *Ambrosiella/Xylosandrus* clade are polyphagous in terms of the tree host taxonomic identity, which contributes to their success as invasive species in many parts of the world [62, 63]. They are, however, highly specific to a particular stage of the tree tissue, death, and decay. This ecological niche—freshly dead or live but internally compromised tree branches—conforms to that of the necrotrophic phytopathogenic relatives of *Ambrosiella*, and it seems to have driven the adaptation of the beetle’s semiochemical ecology and host-selection behavior [64]. The *Meredithiella* lineage, associated with various Corthylini beetles, shows similarly high mutual fidelity. However, *Meredithiella* fungi appear to be more specialized in terms of tree host range, as many species are found in specific hardwood tree species [65]. Testing the extent to which these joint ecologies are driven by the fungal or the beetle ancestral ecology will require a more comprehensive sample of phylogenetic relatives of both the fungi and the beetles. Genome sequences of *Wolfgangiella, Phialophoropsis*, and *Toshionella* remain unavailable.

The contrasting host specificity between *Ophiostomataceae* and *Ceratocystidaceae* highlight the diversity of evolutionary strategies in ambrosia symbioses, with *Ophiostomataceae* retaining a more flexible, promiscuous relationship to both beetles and trees, whereas *Ceratocystidaceae* shows specialized ecologies.

### Adaptability and promiscuity in *Nectriaceae* ambrosia fungi

In *Nectriaceae*, our phylogenomic analysis revealed that the *Neocosmospora* ambrosia clade (henceforth NAC, formerly known as the Ambrosia *Fusarium* clade or AFC) diverged approximately 9.4 Ma (95% HPD: 5.8–15.1 Ma), aligning closely with the previous estimation (~19.3 Ma; 95% HPD: 10.7–28.2 Ma) in O’Donnell et al. and the divergence of their primary hosts, the *Euwallacea* beetles (~12 Ma) [61]. Although this near-synchronous divergence might suggest a coevolutionary relationship, our findings paint a more complex picture.

Like *Ambrosiella*, the NAC fungi show specificity to the stage of tree death rather than tree taxonomy. The few studied species are parasites on stressed but living trees or colonizers of moribund tissues, but rarely they colonize wood in advanced stages of death and decay [66, 67]. They are able to associate with multiple beetle vectors colonizing the same type of tree tissue. For instance, in Taiwan, NAC species such as AF-18 were found to be associated with three different *Euwallacea* species [Polyphagous Shot Hole Borer (PSHB), Tea Shot Hole Borer, and Kuroshio Shot Hole Borer], whereas individual beetle species like PSHB were found to vector multiple NAC species (AF-13 to AF-18, and *Neocosmospora kuroshium*) [67]. This contrasts with observations from invaded areas, where initial reports suggested strict associations (e.g. *N. euwallaceae* with *Euwallacea fornicatus* and *N. kuroshium* with *Euwallacea kuroshio*). Our results suggest that these apparent strict associations may reflect founder effects rather than inherent specificity.

The capacity to colonize partially living trees appears to be a native ecology of some, but not all, *Neocosmospora/Euwallacea* pairs [68]. These species have become significant invasive pests worldwide, causing substantial damage to both native and cultivated trees [69, 70]. For example, *E. fornicatus* in California and Israel can attack over 200 woody plant species from 58 families, with about 32 species serving as reproductive hosts. The damage by these invasions may be partly due to the ability of their NAC symbionts to colonize and thrive in diverse tree species, although the tree death is attributable to the accumulation and persistence of attacks rather than fungal pathogenicity.

### Recent divergence of *Irpicaceae* ambrosia fungi

A unique case is presented by *I. subulatus* (*Irpicaceae*, formerly known as *Flavodon subulatus* or *Flavodon ambrosius*), which diverged approximately 1.9 Ma (95% HPD: 1.6–54.2 Ma) according to phylogenomic analysis. Although this broad confidence interval overlaps with the origin of its associated beetle hosts in the genera *Ambrosiodmus* and *Ambrosiophilus* around 19 Ma [61]. This temporal discrepancy suggests an asynchronous adaptation of the beetle and the fungus, potentially involving beetle lineages feeding on free living wood-decay fungi, later followed by a delayed evolution of the fungal adaptations to dispersal via the insect vector. It may also be due to an undersampled phylogeny, reflecting the fact that basidiomycete ambrosia fungi are poorly sampled and virtually unstudied, even though some are associated with the most basal clades of scolytine beetles [71].

*Irpex subulatus* can produce fruiting bodies independent of beetle galleries [72], allowing for aerial spore dispersal similar to how *Termitomyces* fungi achieve horizontal transmission through spores produced from termite nests, although *Termitomyces* remains dependent on termites for colony establishment and growth [73]. This capacity for environmental existence outside of the insect gallery, also observed in other ambrosia fungi like *R. lauricola* [74], may confer ecological advantages, allowing *I. subulatus* to survive without its beetle hosts and colonize new niches, akin to the situation in *Amylostereum*, a basidiomycete in which some populations are dispersed vertically by symbiotic wood-wasp [75], and some are dispersed horizontally [76]. Such trait retention potentially influences the fungus’s population genetics, dispersal capabilities, and evolutionary trajectory, contributing to its success across multiple beetle host species and geographic regions [77, 78]. The apparent evolutionary mismatch between *I. subulatus* and its hosts presents an intriguing scenario for studying the dynamics of symbiont acquisition and coevolution in ambrosia systems, particularly in cases where the symbiont may have been acquired more recently in evolutionary time. Additional ecological, biological, and genomic data on *Irpex* ambrosia fungi from their native range would greatly enhance our understanding of their evolutionary trajectories.

### Genome features of ambrosia fungi show family-specific differences without broad phylogenetic trends

Our PIC analysis revealed that evolutionary transitions to the ambrosia lifestyle were not accompanied by significant changes in most genomic features when accounting for phylogenetic relatedness (Fig. 2 and Table 2). The analysis included genome size, TE coverage, number of genes, number of secreted proteins, and GC content. None of these features showed a significant difference between ambrosia and nonambrosia fungi (Table 2, all *P-*values > .05). These results suggest that contrary to expectations derived from studies of other symbiotic microorganisms [79, 80], there is no clear genomic convergence associated with the ambrosia lifestyle across different fungal lineages. Adaptations to the ambrosial lifestyle might be lineage-specific rather than following a general pattern. This aligns with observations in other symbiotic systems, where genomic changes often reflect the unique evolutionary trajectories and ecological contexts of specific lineages [81–83]. In ambrosia fungi, as in other symbiotic systems, these adaptations may be shaped by factors such as the host tree species, competing microorganisms, and specific environmental conditions [84–86].

**Table 2:** PIC analysis results for genomic features in relation to ambrosia lifestyle.

Trait	Estimate	Std Error	*T*-value	*P*-value
Genome size	1.13E+07	1.07E+07	1.1	.3
TE coverage	9.53E-01	4.61E+00	0.2	.8
Number of genes	1.70E+03	3.88E+03	0.4	.7
GC content	2.98E+00	3.15E+00	0.9	.3
Secret proteins	5.70E+02	1.27E+03	0.5	.7

No significant associations were found after controlling for phylogeny.

### Metabolic versatility and genomic stability in ambrosia fungi

Our functional gene analysis of ambrosia fungi revealed a more moderate evolutionary pattern than typically seen in symbionts. Unlike extreme genome modifications in many symbionts [80, 87–89], ambrosia fungi maintained nearly equivalent mean gene copy numbers (3.51) compared to non-ambrosia fungi (3.52), aligning with observations in other eukaryotic symbionts like mycorrhizal fungi [8, 80] and lichen mycobionts [90].

**Figure 3:**
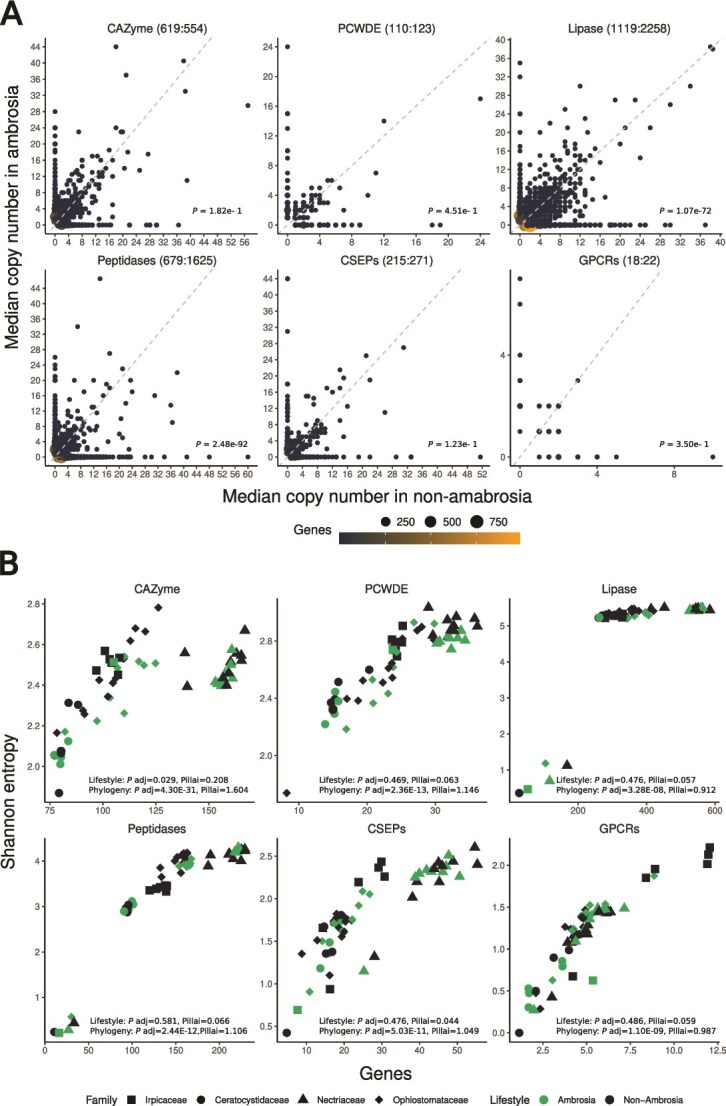
Gene repertoire dynamics in ambrosia fungi. (A) Gene copy number comparison between ambrosia and nonambrosia fungi. Each point represents a gene family, with its position indicating mean copy numbers in ambrosia (*y*-axis) and nonambrosia (*x*-axis) fungi. Color intensity and dot size reflect the number of genes at that ratio. Dashed line represents equal copy numbers. Inset values show total gains and losses per functional group in ambrosia fungi (gain:loss). (B) Functional diversity analysis. Each point represents a genome, with *x*-axis showing gene count and *y*-axis showing Shannon entropy diversity. Green dots indicate ambrosia fungi, black dots indicate nonambrosia fungi. Point shapes denote fungal families: squares for *Irpicaceae*, circles for *Ceratocystidaceae*, triangles for *Nectriaceae*, and diamonds for Ophiostomataceae. *P-*values and Pillai’s trace denote significance of diversity differences between ambrosia lifestyle (lifestyle) or fungal family (phylogeny) for each gene category.

Specific gene families varied between fungal clades (Fig. 3A). CAZymes showed a slight increase in ambrosia fungi (gain/loss ratio 1.12), while Plant Cell Wall Degrading Enzymes (PCWDEs) showed near-balance to slight reductions (gain/loss ratio 0.89), suggesting adaptation to simpler carbohydrates versus complex wood polymer degradation. This indicates refinement rather than substantial decreases of these enzyme families, contrasting with extensive biosynthetic pathway losses in other symbionts [81, 89, 91]. GPCRs showed near-balance to slight reduction (gain/loss ratio 0.818), reflecting ambrosia fungi's dual nature as symbionts retaining many characteristics of free-living relatives. The absence of Pth11-like GPCRs suggests maintained environmental sensing capabilities, though our data can't determine whether these relate to interactions with host tree tissues or beetle mycangia. Lipases and peptidases showed more widespread reductions (gain/loss ratios 0.495 and 0.418), primarily involving low-copy-number genes (Supplementary Table S4), resembling gene family contractions in other eukaryotic symbionts.

Correlation tests between functional gene richness and diversity (Fig. 3B) revealed phylogenetic relatedness had much greater influence than ambrosial lifestyle. Only CAZymes showed significant lifestyle-related difference (*P* = 0.006), suggesting potential impact on carbohydrate metabolism. For other gene categories, lifestyle didn't significantly influence functional diversity (*P* > 0.05). Phylogenetic effects (Family: Pillai = 0.912-1.604, *P* < 1.10E-08) consistently outweighed lifestyle effects (Ambrosia: Pillai = 0.044-0.342, *P* = 0.029-0.581). This variability in functional diversity aligns with the observation that eukaryotic symbionts often show more variable patterns of gene richness modification compared to prokaryotic symbionts [9, 92–94].

The retention of genome size and metabolic diversity in ambrosia fungi likely relates to their ecological requirements in wood substrate. Unlike internal symbionts, ambrosia fungi face constant cycles of colonization and competition in the ephemeral moribund wood while maintaining their symbiotic relationship with beetles. This requires maintaining diverse metabolic capabilities [2, 13] to quickly establish dominance, outcompete other fungi [95], and utilize varied resources. Moreover, the need to re-enter the mycangia of dispersing adult beetles adds another layer of selective pressure [27, 29]. Ambrosia fungi must not only thrive in the gallery environment but also maintain their ability to grow in a form suitable for mycangial transport. The genomic “refinement” of ambrosia fungi can be viewed as an adaptive strategy balancing metabolic versatility with symbiotic specialization. This evolutionary trajectory allows them to remain competitive while meeting symbiotic demands, demonstrating that obligate symbiosis does not always lead to extreme genome modification, especially in variable environments.

### Differential evolution of CAZyme families in ambrosia fungi

**Figure 4:**
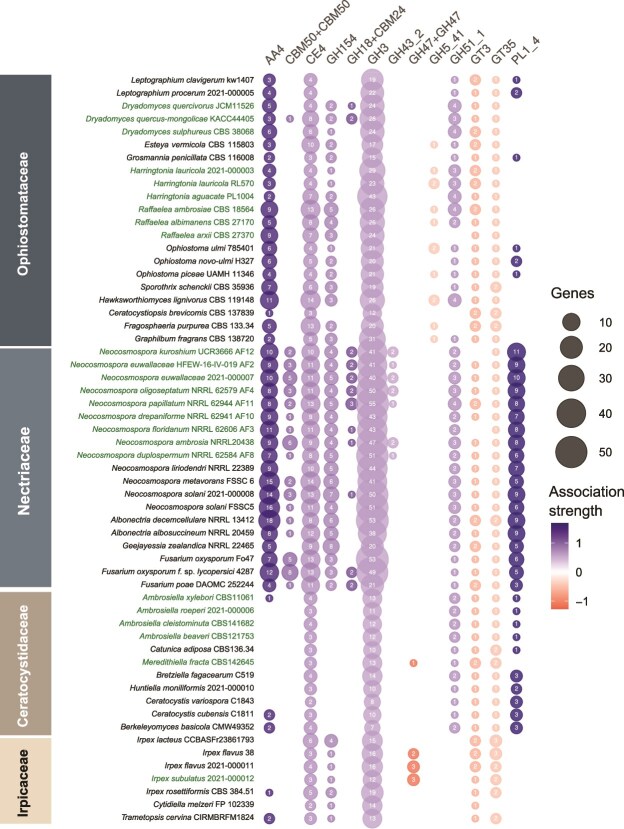
Bubble plot illustrating patterns of CAZyme enrichment and depletion across ambrosia fungi (green) and their nonambrosia relatives (black). CAZyme families shown are significantly associated with ambrosia lifestyle (*P* < .05). Bubble size represents gene count per species. *X*-axis: CAZyme families, color-coded by association direction (blue: positive, red: negative), with color intensity indicating association strength. *Y*-axis: fungal species in the PIC test, ambrosia lineages are highlighted in green.

Our PIC analysis revealed family-specific enzyme evolution patterns in ambrosia fungi rather than uniform trends across lineages. We identified significant associations between specific CAZyme families and ambrosia lifestyle, with distinct expansion/contraction patterns across different fungal families, highlighting the importance of considering phylogeny when interpreting results (Fig. 4 and Supplementary Table S5).

The AA4 family (vanillyl-alcohol oxidases for lignin modification) showed significant expansion correlated with ambrosia lifestyle (Estimate = 1.61, *P* = 0.011), primarily driven by non-ambrosial lineages within Nectriaceae, particularly Neocosmospora species. This suggests possible metabolic versatility expansion followed by contraction in insect-vectored lineages.

The CE4 family (acetyl xylan esterases for hemicellulose modification) displayed significant expansion (Estimate = 0.81, *P* = 0.005), particularly in Ophiostomataceae, suggesting enhanced hemicellulose modification capabilities may be especially important for these ambrosia fungi [96], possibly reflecting different substrate preferences or gallery construction strategies.

The GH3 family (β-glucosidases for cellulose degradation) showed significant expansion (Estimate = 0.65, *P* = 0.046), more prominently in both Ophiostomataceae and Ceratocystidaceae ambrosia lineages, suggesting convergent evolution toward more efficient cellulose processing for better nutrient extraction and wood manipulation.

Signals from Irpicaceae ambrosia lineages were relatively weak in our analysis, with only one genome included. Our results highlight the diverse nature of ambrosia symbiosis across fungal lineages, suggesting they've retained or evolved specialized enzymatic toolkits optimized for unique ecological niches rather than uniformly losing or gaining wood-degrading functions [97].

### RIP activity and genomic adaptation in ambrosia fungi

**Figure 5:**
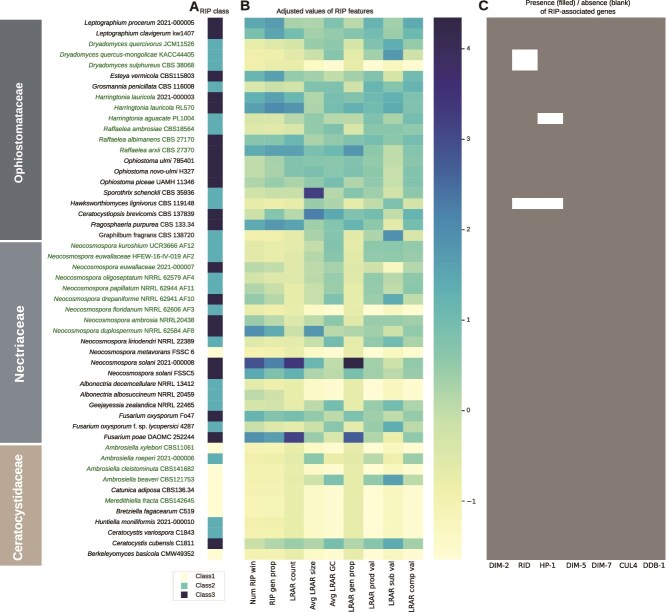
Genome-wide RIP analyses of ambrosia (green) and nonambrosia (black) fungi. (A) RIP classes assigned based on the proportion of the genome affected by RIP, ranging from Class 1 (0%–0.2%) to Class 3 (1%–5%) [42]. (B) Heatmap of standardized values for key RIP features: Number of RIP affected windows (Num_RIP_win), RIP affected genomic proportion (%) (RIP_gen_prop), count of LRARs (LRAR_count), average size of LRARs (bp) (Avg_LRAR_size), average GC content of LRARs (%) (avg_LRAR_GC), genomic proportion of LRARs (bp) (LRAR_gen_prop), product value for LRARs (LRAR_prod_val), substrate value for LRARs (LRAR_sub_val), and composite value for LRARs (LRAR_comp_val). (C) Presence (filled) or absence (blank) of known RIP-associated genes identified by blast against *Neurospora crassa* Shear & B.O. Dodge homologs. *Irpicaceae* species were excluded as RIP is primarily observed in Ascomycota.

RIP mutations do not differ overall between ambrosia and nonambrosia fungi (Fig. 5). Statistical analysis showed no difference in RIP-affected windows (Mann–Whitney *U*-test, *P* = .981), RIP-affected genomic proportion (*P* = .881), or count of LRARs (*P* = .859). This lack of statistical difference suggests that ambrosia lifestyle alone is not a primary determinant of RIP activity.

Most fungi in our dataset possessed the key methyltransferases DIM-2 and RID, as well as several cofactors involved in DNA methylation and heterochromatin formation, regardless of their ecology. Only *Dryadomyces quercus-mongolicae* and *Didymocarpus sulphureus* lacked a detectable RID gene, whereas the free-living relative *Hawksworthiomyces lignivorus* lacked both RID and HP-1. The retention of these genes across most species, regardless of their ambrosia status or observed RIP activity, suggests that factors beyond symbiotic lifestyle influence the maintenance of these genomic elements. However, the presence of these genes alone does not guarantee RIP activity [98]. Their maintenance in ambrosia fungi could indicate potential for cryptic sexual cycles [25, 26], additional gene functions beyond repeat silencing, or recent loss of RIP activity with insufficient time for gene loss.

## Conclusion

These findings reveal diverse molecular adaptations in ambrosia fungi-beetle symbioses, with lineage-specific strategies more sophisticated than previously recognized. Our genomic analyses of virtually all available ambrosia fungal genomes show subtle adaptations, though domestication extent remains unclear. Future research should focus on biochemical characterization within specific lineages and comparative analysis. Improved genome assemblies and experimental data like lifecycle transcriptomes will enhance understanding of mutual signaling, nutrition provisioning, and specificity mechanisms in these fascinating ecological relationships.

## Supplementary Material

Supplementary_material_wraf039

## Data Availability

Raw sequences associated with this paper have been deposited with links to Bioproject accession number PRJNA1084195 in the NCBI BioProject database (https://www.ncbi.nlm.nih.gov/bioproject/). All code is available at https://github.com/ythuangmyco/AmbrosiaFungiEvoGenomics.git
