# Supplementary material for: Genome diversification of symbiotic fungi in beetle-fungus mutualistic symbioses
Source: ISME J. 2025 Feb 27;20(1):wraf039. doi: 10.1093/ismejo/wraf039 (PMC13322289; doi:10.1093/ismejo/wraf039)
Supplement: Supplementary_material_wraf039 [file supplementary_material_wraf039.zip › Genome_diversification_of_ambrosia_fungi_Suppl_Legend_wraf039.docx]

Table S1. Fungal isolates used to generate genomic data in this study

Table S2. Genome features of species sequenced in present study. Genomes sequenced in this study are indicated in bold

Table S3. Gene concordance factor (gcf) and site concordance factor (scf) analysis results

Table S4. Gene count distribution in orthogroups of ambrosia and non-ambrosia fungi

Table S5. Results of applying phylogenetically independent contrasts (PIC) to CAZyme families, showing significant expansions of predicted CAZyme families linked to the ambrosia lifestyle

Figure S1. Ancestral state reconstruction of ambrosia fungi. (a) maximum-likelihood method. (b) stochastic character mapping method
