# Supplementary figures and images for: Genome diversification of symbiotic fungi in beetle-fungus mutualistic symbioses
Source: ISME J. 2025 Feb 27;20(1):wraf039. doi: 10.1093/ismejo/wraf039 (PMC13322289; doi:10.1093/ismejo/wraf039)

(A)

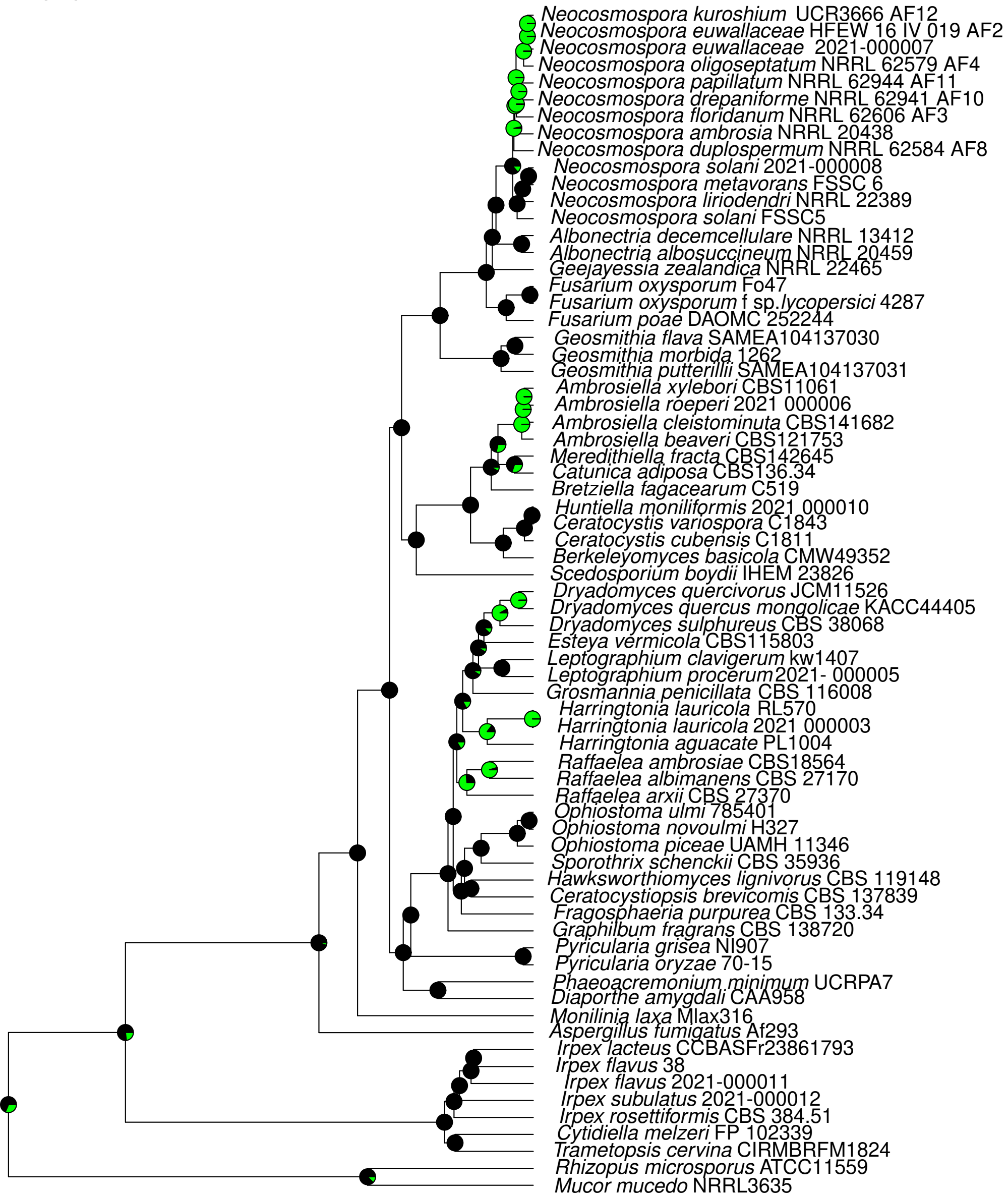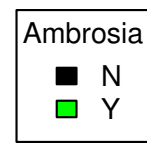

(B)

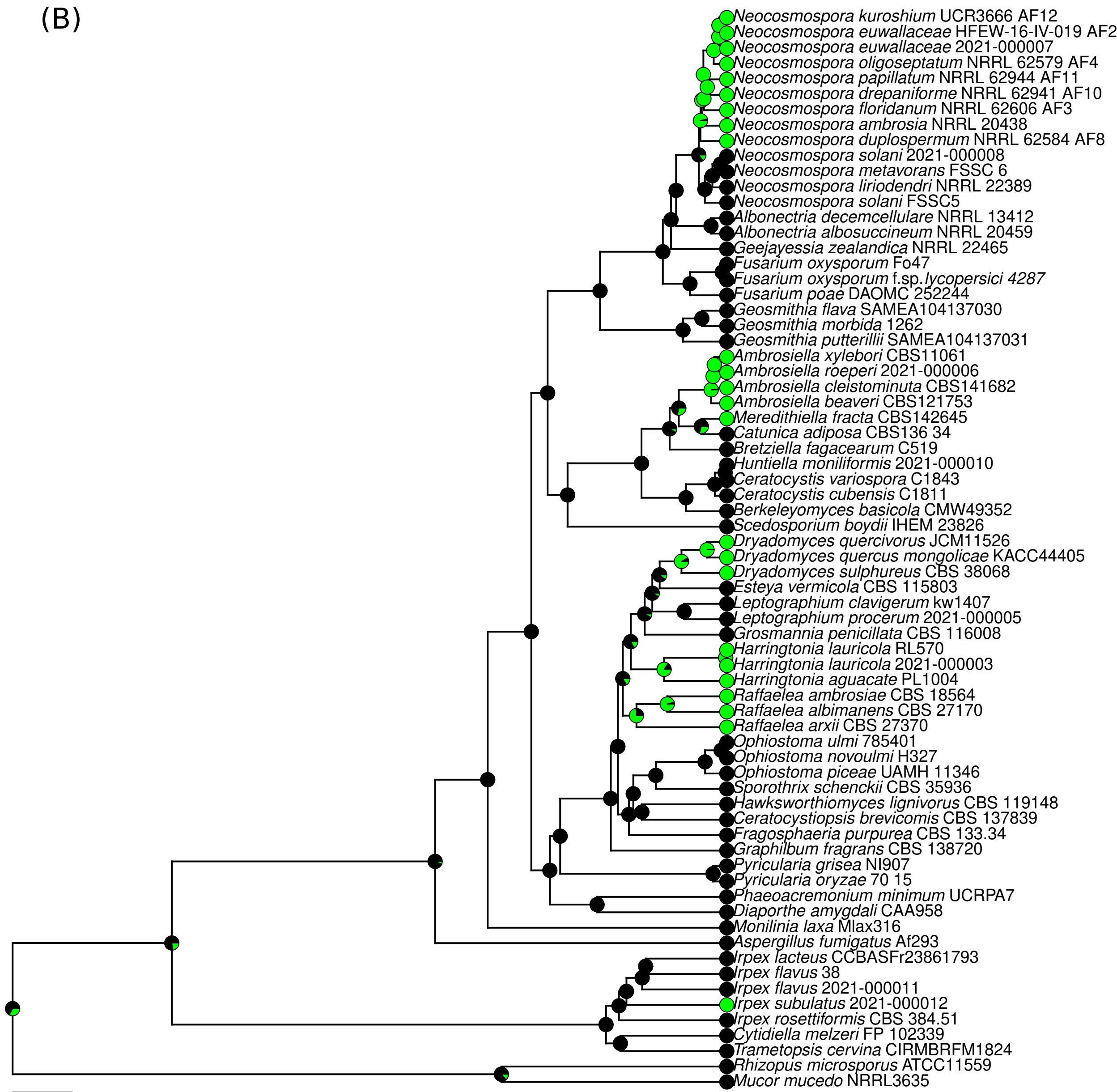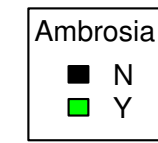

Supplement: Supplementary_material_wraf039 [file supplementary_material_wraf039.zip › FigureS1_R4_wraf039.pdf]
